# Breast cancer colonization by *Malassezia globosa* accelerates tumor growth

**DOI:** 10.1128/mbio.01993-24

**Published:** 2024-09-05

**Authors:** Miao-Miao Liu, Hui-Hui Zhu, Jie Bai, Zi-Ye Tian, Yu-Jing Zhao, Teun Boekhout, Qi-Ming Wang

**Affiliations:** 1 School of Life Sciences, Institute of Life Sciences and Green Development, Hebei University, Baoding, Hebei, China; 2 College of Sciences, King Saud University, Riyadh, Saudi Arabia; 3 Hebei Basic Science Center for Biotic Interaction, Hebei University, Baoding, Hebei, China; 4 Engineering Research Center of Ecological Safety and Conservation in Beijing-Tianjin-Hebei (Xiong’an New Area) of MOE, Xiong’an, China; McMaster University, Hamilton, Ontario, Canada

**Keywords:** breast cancer, *Malassezia*, fungi, IL-17A, tumor-associated macrophage, Sphk1

## Abstract

*Malassezia globosa* is a lipophilic *basidiomycetous* yeast that occurs abundantly in breast tumors and that may contribute to a shortened overall survival of breast cancer (BRAC) patients, suggesting that the yeast may participate in the carcinogenesis of BRAC. However, the mechanisms involved in the *M. globosa*-based acceleration of BRAC are unknown. Here, we show that *M. globosa* can colonize mammary tissue in 7,12-dimethylbenz[a] anthracene-induced mice. The abundance of *M. globosa* shortened the overall survival and increased the tumor incidence. Transcriptome data illustrated that IL-17A plays a key role in tumor growth due to *M. globosa* colonization, and tumor-associated macrophage infiltration was elevated during *M. globosa* colonization which triggers M2 polarization of macrophages via toll-like receptors 4/nuclear factor kappa-B (Nf-κB) signaling. Our results show that the expression of sphingosine kinase 1 (Sphk1) is increased in breast tumors after inoculation with *M. globosa*. Moreover, we discovered that Sphk1-specific small interfering RNA blocked the formation of lipid droplets, which can effectively alleviate the expression of the signal transducer and activator of the transcription 3 (STAT3)/Nf-κB pathway. Taken together, our results demonstrate that *M. globosa* could be a possible factor for the progression of BRAC. The mechanisms by which *M. globosa* promotes BRAC development involve the IL-17A/macrophage axis. Meanwhile, Sphk1 overexpression was induced by *M. globosa* infection, which also promoted the proliferation of MCF-7 cells.

**IMPORTANCE:**

Literature has suggested that *Malassezia globosa* is associated with breast tumors; however, this association has not been confirmed. Here, we found that *M. globosa* colonizes in breast fat pads leading to tumor growth. As a lipophilic yeast, the expression of sphingosine kinase 1 (Sphk1) was upregulated to promote tumor growth after *M. globosa* colonization. Moreover, the IL-17A/macrophages axis plays a key role in mechanisms involved in the *M. globosa*-induced breast cancer acceleration from the tumor immune microenvironment perspective.

## INTRODUCTION

The genus *Malassezia* (*Basidiomycota*, *Malasseziomycetes*) is a group of lipophilic yeasts that resides on human and animal skin sites representing >90% of the fungus in the skin mycobiome (1). In general, *Malassezia* yeasts are usually associated with a variety of dermatological diseases under certain conditions, e.g., pityriasis versicolor, dermatitis, and dandruff (2). Several recent studies have shown that *Malassezia* plays a key role in promoting various cancers, including pancreatic cancer, breast cancer (BRAC), and skin cancer, in addition to the skin diseases they usually cause (3–5). *Malassezia* has been reported to produce multiple proinflammatory biological properties that contribute to tumor initiation, such as enrichment of inflammatory factors, degradation of the extracellular matrix, and destruction of the epithelial barrier (6). As a lipophilic yeast, *Malassezia* plays a role in the development of skin cancer as some lipid breakdown products activate components of the tumor-promoting pathways (7). Furthermore, the other case of *Malassezia* being associated with cancer is the involvement of *Malassezia globosa* in pancreatic cancer. Aykut et al. demonstrated that *M. globosa* accelerated pancreatic oncogenesis by mannose-binding lectin. However, once *M. globosa* was excised in pancreatic tumor-bearing mice, its prognosis improved (4). Recent research proved that *Malassezia* species are also abundantly present in breast tumors (8). Narunsky-Haziza et al. found significantly shorter overall survival (OS) in BRAC patients with the intra-tumoral presence of *M. globosa* (9). These observations suggest that patients with breast tumors have higher numbers of *M. globosa* that, in turn, may lead to a worsened prognosis of patients.

BRAC remains one of the most diagnosed female cancers worldwide and is the leading cause of all types of cancer-related deaths in women (10). Although the role of proinflammatory cytokine interleukin-17A (IL−17A) in malignancy contradicts existing data, some research shows that IL-17A could promote tumor growth (11, 12). For example, previous reports show that IL-17A accelerates tumor growth in cervical cancer, probably by stimulating angiogenesis (13). Meanwhile, IL-17A has been associated with BRAC as it regulates the activities of nuclear factor kappa-B (Nf-κB) and increases the expression of IL-6 and cyclooxygenase-2 (COX-2) (14). Infections caused by fungi, bacteria, and parasites can induce the production of IL-17A, leading to chronic inflammatory responses (15, 16). Thus, we hypothesize that *M. globosa* colonization in BRAC accelerates IL-17A expression and results in the chronic inflammatory response in the tumor immune microenvironment (TME). Inflammatory responses involve a variety of molecules in carcinogenic processes and corresponding signaling pathways, thus increasing the risk of cancer (17). Research has revealed that the TME is also involved in the pathogenesis of microbial colonized cancer (18). Macrophages are key components of the TME that promote cancer cell migration, invasion, and angiogenesis leading to cancer progression (19). IL-17A plays a role in the activation of different immune cells, including macrophages. Findings by Wang et al. revealed that *Candida albicans* promoted oral cancer development via the IL-17A/macrophage axis (20). Evidence from these studies suggests that tumor-associated macrophages (TAMs) promote tumor metastasis by releasing multiple cytokines, including inflammatory factors, chemokines, and growth factors (21). A widely accepted classification defines macrophages as M1, which are regarded as anti-tumor activity, or M2, which mainly promotes tissue remodeling and tumor progression (22). Nishikawa et al. reported that macrophages may acquire M2 properties that adapt to the inflammatory colon microenvironment under the influence of IL-17A (23). Furthermore, Li et al. reported that gut microbiota-stimulated cathepsin K in human colorectal cancer tissues is always accompanied by high M2 polarization of TAMs in the stroma (24). Herein, we speculate that *M. globosa* can cause macrophages to polarize toward M2 by increasing the expression of IL-17A.

As a lipophilic and commensal yeast, *M. globosa* encodes various enzymes, such as esterases, lipases, lipoxygenases, and proteases, that play a potential role in regulating host interactions and the lipid present directly affects immune-suppressive (25). Phospholipase activity of *Malasszia* spp. catalyzes the production of arachidonic acid (AA), which may elicit inflammatory responses (26). The mammary gland showed a strong capacity to synthesize and secrete lipids, perhaps providing a suitable environment for *M. globosa* residency (27). Therefore, we suspect that *M. globosa* infection leading to lipid metabolism disorder and inflammatory responses is also a contributor to the growth of breast tumors. Considering the close association between *M. globosa* and BRAC, and the unrevealed relationship between *M. globosa* and TME, this study investigated the role of *M. globosa* in stimulating BRAC development and revealed the underlying mechanisms from the perspective of TME in both *in vivo* and *in vitro* experiments.

## RESULTS

### *M. globosa* colonization decreased OS in 7,12-dimethylbenz[a] anthracene-induced BRAC mice

**Fig 1:**
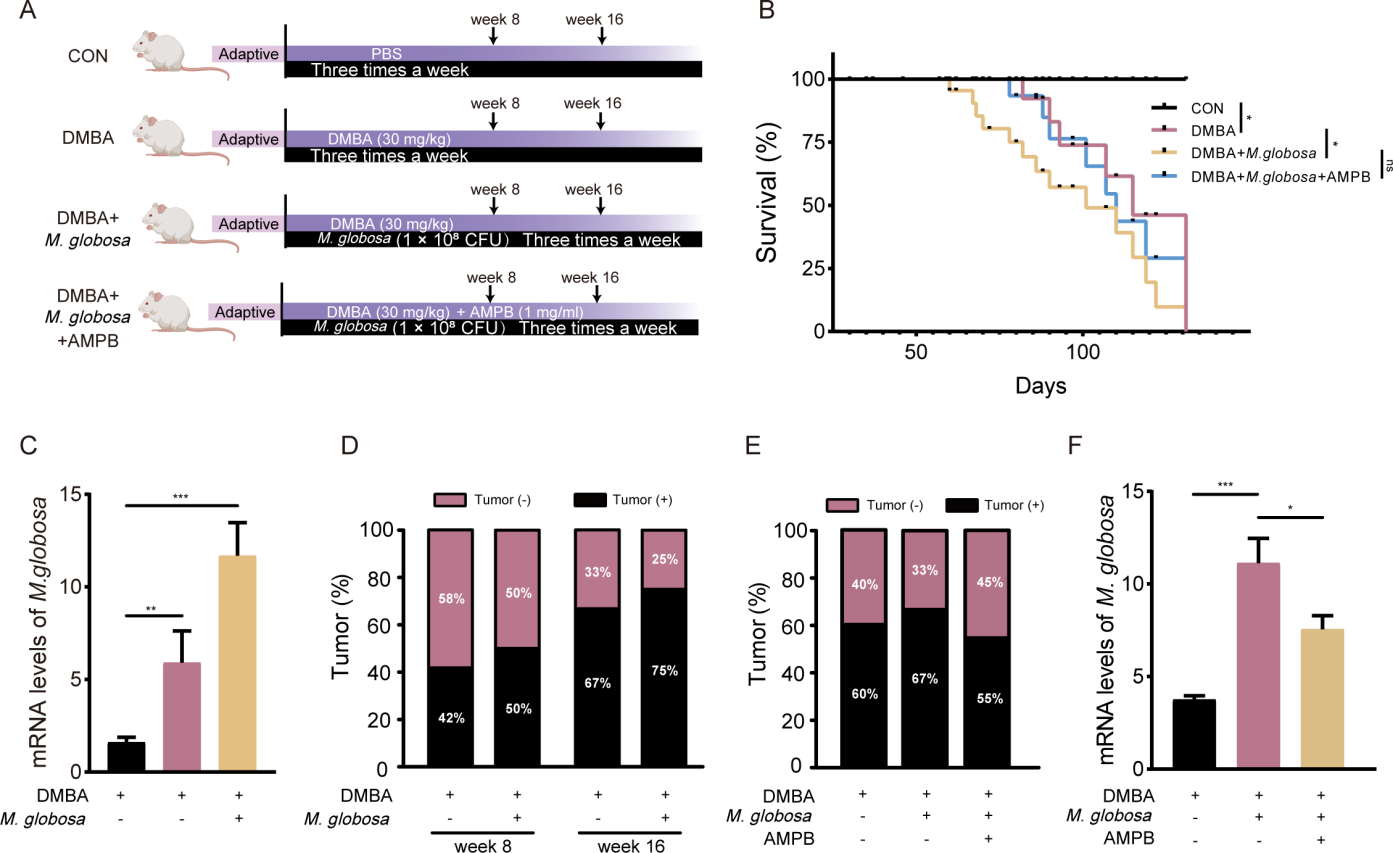
*M. globosa* colonization accelerated breast cancer progress. (**A**) Schematic figure of DMBA-induced mice with *M. globosa* colonization. (**B**) Overall survival for the CON, DMBA, DMBA + *M. globosa*, and DMBA + *M. globosa* + AMPB group. (**C**) The mRNA expressions of *M. globosa* were measured by qPCR. (**D**) Breast tumor incidence in weeks 8 and 16, respectively. (**E**) Breast tumor incidence in week 12 with and without treatment with AMPB. (**F**) The mRNA levels of *M. globosa* with and without treatment with AMPB. Data are mean values ± SD. *P < 0.05, **P < 0.01, and ***P < 0.001.

7,12-Dimethylbenz[a] anthracene (DMBA) was administered to induce BRAC in mice, and then *M. globosa* was injected into a mammary fat pad. As expected, after *M. globosa* colonization mice (DMBA + *M. globosa* group) showed lower OS compared with the mice in the DMBA group (Fig. 1B). Furthermore, the mammary from the DMBA group and DMBA + *M. globosa* group showed more enrichment of *M. globosa* compared with the vehicle (VEH) group. The content of *M. globosa* in the DMBA + *M. globosa* group increased by 1.98-fold compared to the DMBA group (Fig. 1C). Noteworthy, the incidence of breast tumors in the DMBA + *M. globosa* group was higher than in the DMBA group after both 8 and 16 weeks (Fig. 1D). The level of *M. globosa* and tumor incidence decreased after treatment with amphotericin B (AMPB; Fig. 1E and F). However, treatment with AMPB reversed the tumor incidence development but had no significant effect on OS (Fig. 1B and E).

### *M. globosa* colonization promoted the proliferation of BRAC

**Fig 2:**
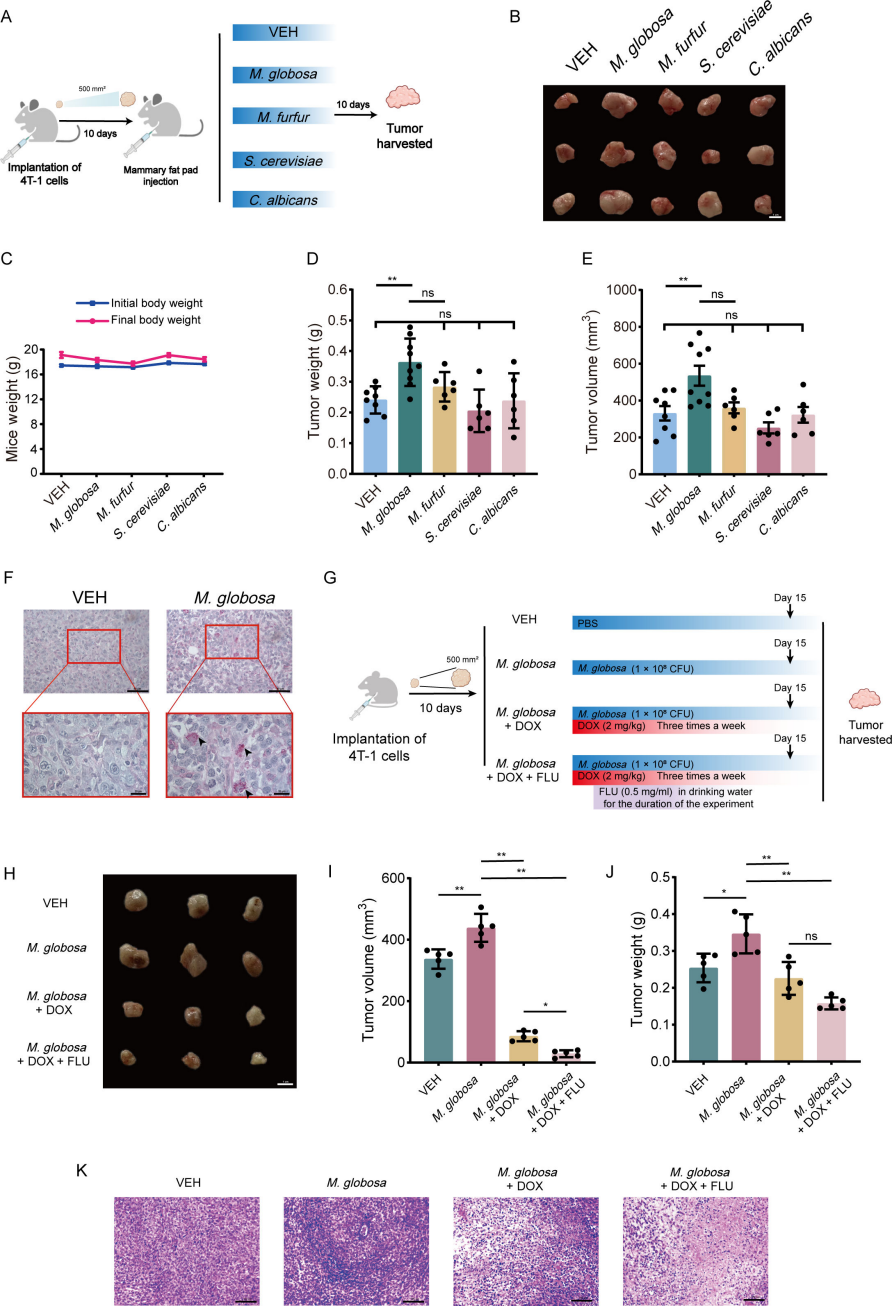
Colonization with *M. globosa* in mammary fat pad promotes breast tumor growth. (**A**) Schematic diagram of specific fungus colonization with mammary fat pad. (**B**) Photographs of orthotopic breast tumor. BALB/c mice were repopulated with vehicle, *M. globosa*, *M. furfur*, *S. cerevisiae*, and *C. albicans* sacrificed at the end of the experiment. Scale bars represent 1 cm. (**C**) Mice weight of initial and final. (**D and E**) Weight and volume of tumors. (**F**) PAS’s reaction stain of VEH and *M. globosa* colonization group. Scale bars represent 50 and 20 µm. (**G**) Schematic diagram of combination treatment, including VEH, *M. globosa*, *M. globosa* + doxorubicin (DOX), and *M. globosa* + DOX + FLU group. (**H**) Representative photographs of breast tumor. Scale bars represent 1 cm. (**I and J**) Weight and volume of tumors after treatment with alone and combination treatment. (**K**) Representative sections of hematoxylin and eosin staining. Scale bars represent 100 µm. Data are mean values ± SD. *P < 0.05, **P < 0.01, and ***P < 0.001.

To investigate whether the abundance of *M. globosa* accelerates BRAC, an orthotopic tumor transplantation experiment was performed (Fig. 2A). Controls were repopulated with *Malassezia furfur*, *Saccharomyces cerevisiae*, *C. albicans*, and treated with PBS (VEH). Only the *M. globosa* colonized group accelerated tumor growth, whereas the groups with other taxa had no effect (Fig. 2B through E). The antifungals AMPB or fluconazole (FLU) were used to ablate *M. globosa* in tumor tissue to observe whether eliminating *M. globosa* would affect tumor growth (Fig. S2A). The tumor size was found to be decreased and the periodic acid-Schiff (PAS) staining showed that the amount of magenta-colored fungus was reduced after treatment AMPB or FLU, which suggests that the reduction of the *M. globosa* load within the tumor could reduce tumor growth (Fig. S2B through E).

To analyze the role of *M. globosa* in the development of BRAC, *in vitro* experiments were conducted, and *S. cerevisiae* was used as a fungal control. Mouse breast cancer cells (4T-1) and Michigan cancer foundation-7 (MCF-7) cell lines were then stimulated with live *M. globosa* (multiplicity of infection [MOI] = 10) and *S. cerevisiae* (MOI = 10), respectively. To analyze whether *M. globosa* could affect the migration of MCF-7 and 4T-1 cells, a transwell assay was conducted. However, no significant difference was found among untreated MCF-7 cells and those treated with *S. cerevisiae* at 24 h but *M. globosa* stimulation greatly enhanced the migration of MCF-7 and 4T-1 cells (Fig. S1A through I).

Thereafter, the combination treatment of FLU and doxorubicin (DOX) enabled tumor regression in *M. globosa* colonized mice (Fig. 2G). The combined treatment of FLU and DOX showed a lower tumor volume compared to the DOX treatment group alone (Fig. 2H through K). The use of combined anticancer drugs and DOX leads to better therapeutic effects.

### TAM accumulation was associated with IL-17A during *M. globosa* colonization

**Fig 3:**
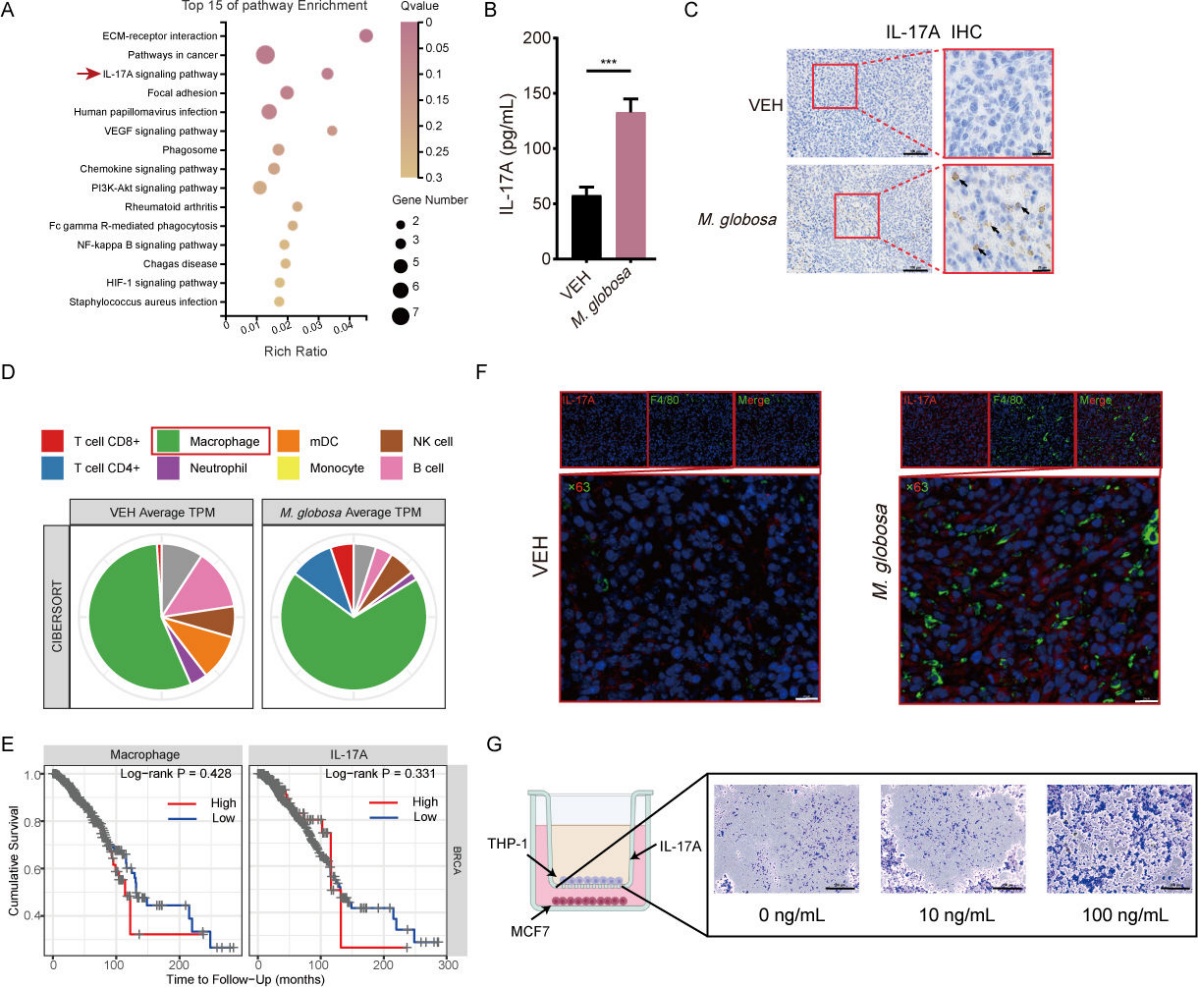
Macrophage infiltration was correlated with the IL-17A signaling pathway in *M. globosa* colonization breast tumor. (**A**) Kyoto Encyclopedia of Genes and Genomes (KEGG) enrichment analysis showed the top 15 enrichment pathways. (**B**) The levels of IL-17A in VEH and *M. globosa* group. (**C**) Representative IHC stain of IL-17A. Scale bars represent 100 and 20 µm. (**D**) Tumor immune cell infiltration based on the mRNA-seq data. (**E**) The IL-17A expression level and macrophage infiltration level in OS of BRAC patients. (**F**) Representative immunofluorescence images of F4/80 (green) and IL-17A (red) expression in the VEH and *M. globosa* group. Scale bars represent 20 µm. (**G**) Effects of transwell migration experiment. Scale bars represent 200 µm. Data are mean values ± SD. *P < 0.05, **P < 0.01, and ***P < 0.001.

According to RNA sequencing analysis, the IL-17A signaling pathway was upregulated in *M. globosa* colonized mice (Fig. 3A). Using immunohistochemistry (IHC) and enzyme-linked immunosorbent assay (ELISA), we measured the expression of IL-17 that was upregulated after colonization with *M. globosa in vivo* (Fig. 3B and C), which is consistent with the results obtained *in vitro* (Fig. S3F). Meanwhile, the colonization of *M. globosa* leads to the activation of the IL-17A signaling pathway, which stimulates the production of various inflammatory cytokines and chemokines *in vivo*, including IL-1β, IL-6, IL-23, and tumor necrosis factor α (TNF-α) (Fig. S3D and E). Herein, we found that *M. globosa* leads to an increase of intracellular IL-1β, IL-6, IL-23, and TNF-α by co-culturing MCF-7 or 4T-1 cells with *M. globosa* (MOI = 10). However, infection with *S. cerevisiae* had no significant change in those inflammatory cytokines ( Fig. S3G and H).

The RNA-seq data set was used to estimate tumor immune cell infiltration from the ImmuCellAI-mice. We found that macrophages are essential for the tumor-promoting effect of *M. globosa*. Our results showed that the level of macrophage infiltration was upregulated in the *M. globosa*-infected group compared with the VEH group (Fig. 3D; Table S4). Furthermore, overexpression of IL-17A and infiltration of macrophages leads to decreased OS (Fig. 3E), suggesting that both are closely related to the prognosis in BRAC patients from the TCGA (The Cancer Genome Atlas Program; https://www.cancer.gov/ccg/) data in TIMER (Tumor IMmune Estimation Resource, https://cistrome.shinyapps.io/timer/). It is worth noting that the level of macrophage infiltration correlated positively with the expression of IL-17A, which was verified by the immunofluorescence (IF) of F4/80 and IL-17A (Fig. 3F). The excessive enrichment of *M. globosa* in the mammary fat pad may promote the production of IL-17A, thus inducing macrophage infiltration in BRAC. The relationship between IL-17A and macrophage infiltration in BRAC was assessed by a transwell migration experiment (Fig. 3G), and this indicated that MCF-7 cells treated with rhIL-17A attracted more Tohoku hospital pediatrics-1 (THP-1)-derived macrophages into the lower chamber, which means that the overexpression of IL-17A promotes macrophage infiltration.

### *M. globosa* promoted M2 polarization of macrophages

**Fig 4:**
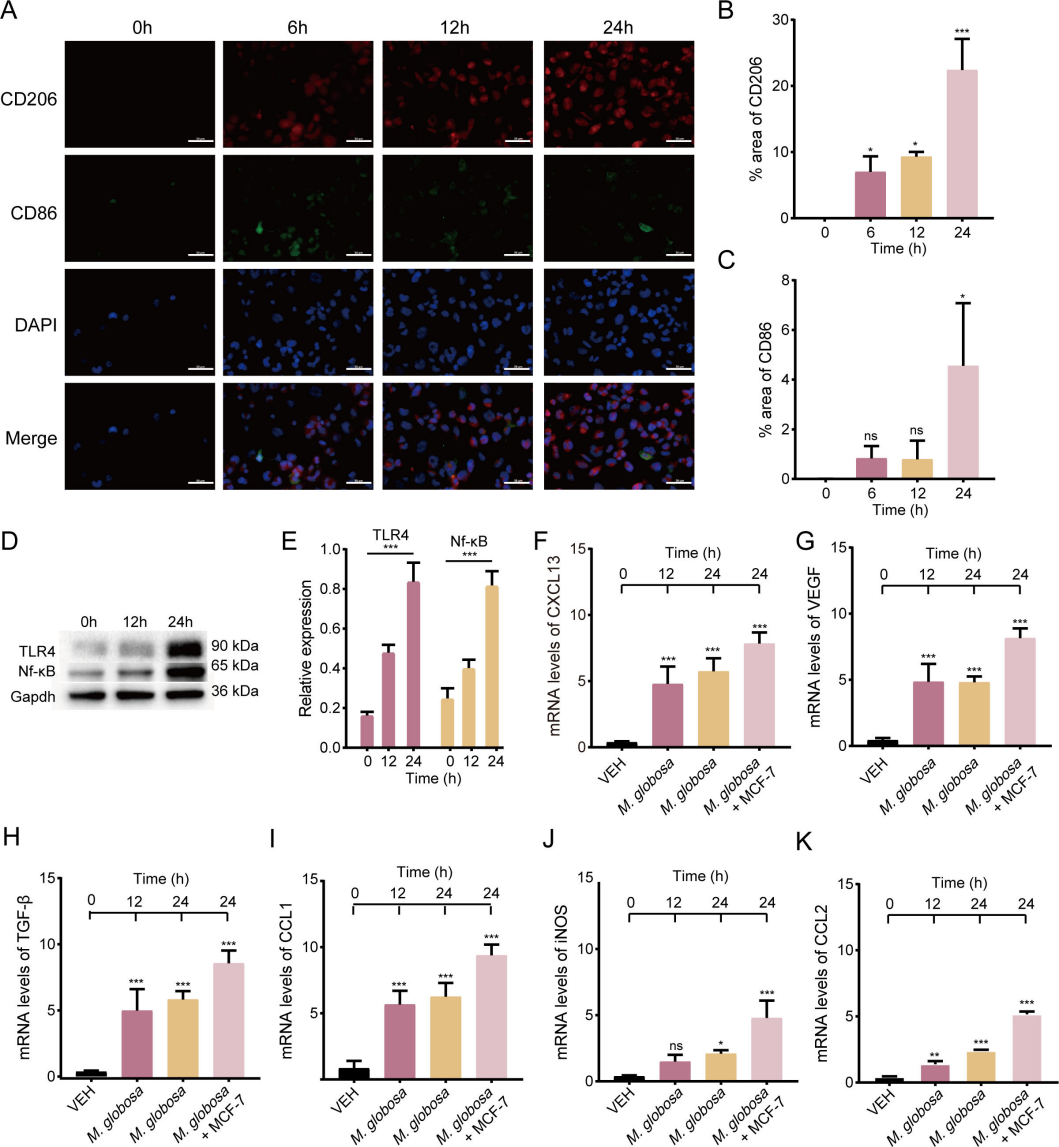
*M. globosa* promoted M2 polarization of macrophages. (**A**) Representative IF images of CD86 (green) and CD206 (red) in THP-1-derived macrophages. Scale bars represent 50 µm. (**B and C**) The fluorescence intensity and area were expressed as a percentage. (**D**) Western blotting assays analyzed the expression of TLR4 and Nf-κB. (**E**) The intensity of TLR4 and Nf-κB was standardized to the protein expression levels of Gapdh. (**F and K**) The mRNA expressions of CCL2, iNOS, CXCL13, VEGF, TGF-β, and CCL1 were measured by qPCR. Data are mean values ± SD. *P < 0.05, **P < 0.01, and ***P < 0.001.

To assess the effect of *M. globosa* on macrophage polarization, we conducted IF staining to measure the marker of M1 (CD86) and M2 (CD206) phenotype macrophages after THP-1-derived macrophages were infected with *M. globosa*. We found that M2 polarization of THP-1-derived macrophages was enhanced after stimulation with *M. globosa* in 12 and 24 h (Fig. 4A through C). Next, we investigated the cytokines secreted by THP-1-derived macrophages treated with *M. globosa* by qPCR. When THP-1-derived macrophages were directly treated with *M. globosa*, both M1-like (i.e., CCL1 and iNOS) and M2-like markers (i.e., CCL2, TGF-β, VEGF, and CXCL13) were found to be upregulated, with M2-like markers being dominant, suggesting *M. globosa*-induced macrophage toward M2 polarization (Fig. 4F through K). Meanwhile, we found that the mRNA levels of M2-like markers were greatly enhanced in macrophages stimulated with the medium produced by the co-culture with *M. globosa* and MCF-7 (Fig. 4F through K).

The previous studies demonstrated that Nf-κB signaling could be induced by toll-like receptors (TLRs); thus, we suspect that Nf-κB signaling could be activated by TLR4 to result in macrophage M2 polarization. We investigated the expression of TLR4 and Nf-κB by western blot. The results showed that the expression of TLR4 and Nf-κB increased at 12 h, and the levels were higher at 24 h (Fig. 4D and E), which means the expression level increased time-dependently.

### Neutralization of IL-17A mitigated the tumor progression promoted by *M. globosa* colonization

**Fig 5:**
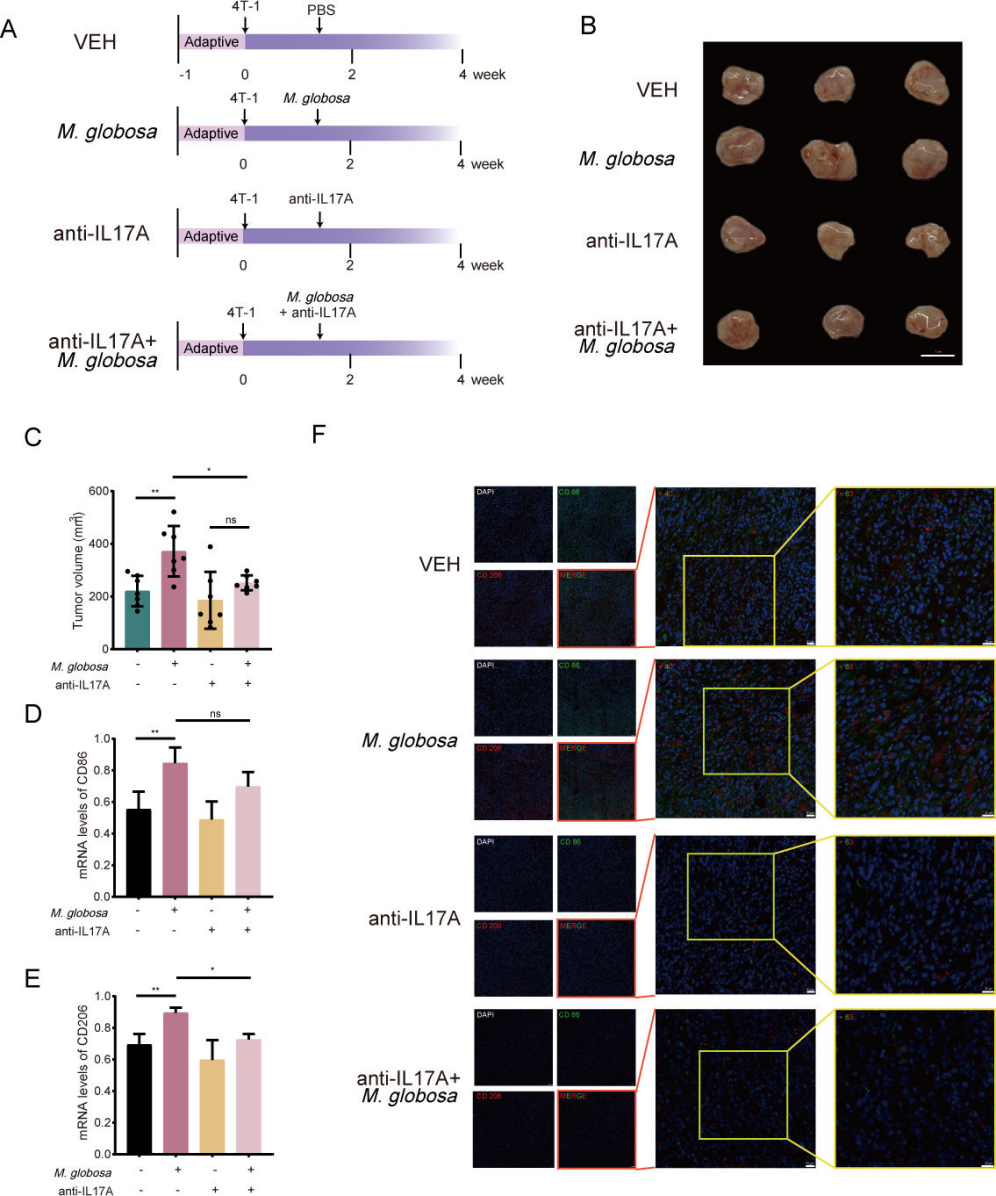
The neutralization of IL-17A mitigated the progression of tumors promoted by the colonization of *M. globosa*. (**A**) Schematic diagram of IL-17A neutralization in mice; VEH, *M. globosa* (colonization with *M. globosa*), anti-IL17A (IL-17A neutralization), and *M. globosa* + anti-IL17A (*M. globosa* colonization with IL-17A neutralization). (**B**) Representative photographs of the tumor. Scale bars represent 1 cm. (**C**) Volume of tumors. (**D and E**) The mRNA levels of CD86 and CD206. (**F**) Representative IF images of CD86 (green) and CD206 (red). Scale bars represent 20 µm. Data are mean values ± SD. *P < 0.05, **P < 0.01, and ***P < 0.001.

The above study demonstrated that *M. globosa* promotes BRAC cell proliferation by IL-17A. Therefore, we wanted to confirm the role of IL-17A in *M. globosa* promoting BRAC *in vivo* (Fig. 5A). Although there was no statistical difference observed between the anti-IL-17A group and anti-IL-17A + *M. globosa* group, the tumor volume in the anti-IL17A + *M. globosa* group was reduced compared with the *M. globosa* group (Fig. 5B and C). Meanwhile, the mRNA expression of CD86 and CD206 was higher after colonization with *M. globosa*, but the relative abundance of M2 (CD206) macrophages decreased after IL-17A neutralization (Fig. 5D and E). Additionally, more M2-like macrophages accumulated in the tumor of mice with *M. globosa* infection, and this was reduced after IL-17A neutralization (Fig. 5F).

### Sphingosine kinases 1 knockdown decreases lipid disorder induced by *M. globosa*

The number of lipid droplets increased significantly in *M. globosa*-stimulated breast tumor tissue when compared with the VEH and *S. cerevisiae* groups (Fig. S5A). COX-2 and 5-lipoxygenase (LOX) play a key role in the metabolization of AA, which is involved in *Malassezia*-caused lipid disorder (28). A significant rise in COX-2 and 5-LOX expression was observed in the *M. globosa* colonized group compared to the VEH and *S. cerevisiae* groups (Fig. S5C through E), indicative of a lipid metabolism disorder induced by *M. globosa*. Similar results were obtained for MCF-7 and 4T-1 cells. The excessive lipid droplet formation in MCF-7 and 4T-1 cells after stimulation with *M. globosa* was confirmed by oil red O staining (Fig. S5F). Based on the types of lipid abnormalities, those can be categorized into total cholesterol (TG), low-density lipoprotein (LDLC), and high-density lipoprotein (HDLC). The result showed that the amount of TG, HDLC, and LDLC increased in *M. globosa*-stimulated MCF-7 and 4T-1 cells (Fig. S5G).

**Fig 6:**
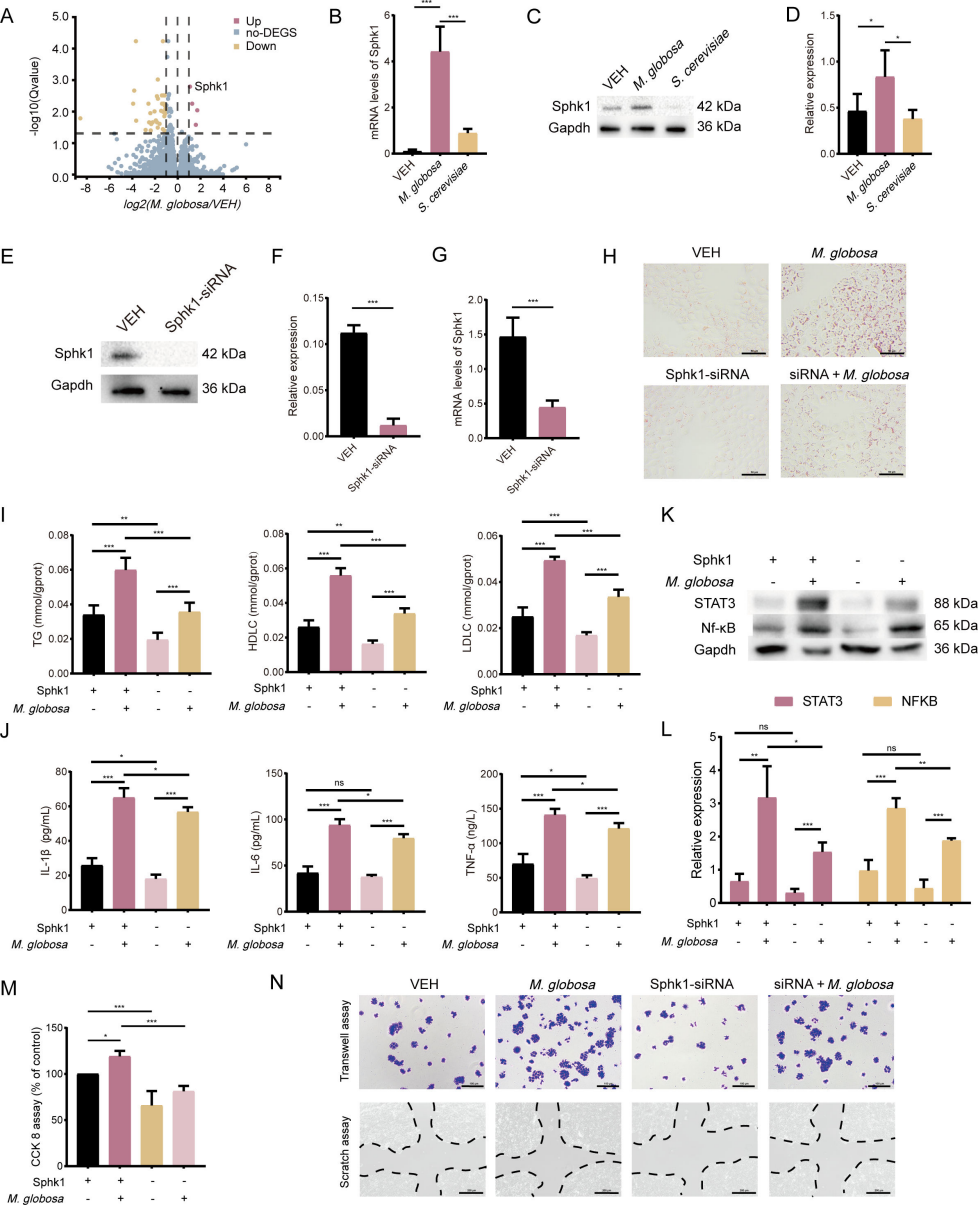
The Sphk1-siRNA interference fragments inhibited lipids *in vitro*. (**A**) Differential gene expressions are visualized in volcano plots. (**B**) The mRNA levels of Sphk1 in colonization with VEH, *M. globosa*, and *S. cerevisiae* in tumor tissue. (**C**) Western blotting assays analyzed the expression of Sphk1. (**D**) The intensity of Sphk1 was standardized to the protein expression levels of Gapdh. (**E and G**) Knockdown of Sphk1 in MCF-7 cells. (**H**) Knockdown of Sphk1 inhibited the formation of lipid droplets. Scale bars represent 50 µm. (**I**) Activities of TG, LDLC, and HDLC. (**J**) The levels of IL-1β, IL-6, and TNF-α after stimulation with *M. globosa* in Sphk1-siRNA interference cell. (**K and L**) Western blotting assays analyzed the expression of STAT3 and Nf-κB. (**M**) The effect of *M. globosa* on Sphk1-siRNA interference cell proliferation. (**N**) Effects of *M. globosa* on transwell assay and scratch assay. Scale bars represent 100 and 200 µm. Data are mean values ± SD. *P < 0.05, **P < 0.01, and ***P < 0.001.

Transcriptome data showed that the expression of sphingosine kinase 1 (Sphk1) was increased after *M. globosa* colonization (Fig. 6A). The mRNA levels of Sphk1 increased in the *M. globosa* group, but the VEH and *S. cerevisiae* groups had no significant change (Fig. 6B). Similar results were found at the protein level as concluded from western blot analysis (Fig. 6C and D). Then, the role of Sphk1 was investigated in *M. globosa*-infected MCF-7 cells. In MCF-7 cell lines, Sphk1 was knocked down to investigate the changes in *M. globosa*-induced intracellular lipids. The expression of Sphk1 was downregulated after Sphk1 small interfering RNA (siRNA) knockdown (Fig. 6E through G). In contrast to the VEH group, the Sphk1-siRNA group exerted inhibitory effects on lipid droplet formation. Then, the MCF-7 cells were co-cultured with *M. globosa*, and the oil red O staining showed that lipid droplet formation was decreased in the siRNA + *M. globosa* group (Fig. 6H). The levels of TG, HDLC, and LDLC also reduced in the siRNA + *M. globosa* group compared with the *M. globosa*-infected group alone (Fig. 6I). In addition, the contents of IL-1β, IL-6, and TNF-α have also decreased in the siRNA + *M. globosa* group compared to *M. globosa* group (Fig. 6J). Sphk1 encourages the activation of signal transducer and activator of transcription 3 (STAT3)/Nf-κB through positive autocrine-loop signaling. The results revealed that the expression of STAT3/Nf-κB signaling pathway in the *M. globosa* group increased, but it has been downregulation in the siRNA + *M. globosa* group (Fig. 6K and L). In addition, the Sphk1-siRNA MCF-7 strain inhibited proliferation and survival in MCF-7 cells, and the cell migration and viability were found to be decreased after reducing the expression of Sphk1 in *M. globosa*-infected MCF-7 cells compared with the *M. globosa* alone group (Fig. 6M and N).

## DISCUSSION

*Malassezia* belongs to the fungal division *Basidiomycota* where it resides in its own class *Malasseziomycetes*, and it plays an important role in the mycobiome of the mammalian skin (29, 30). Recently, a pan-cancer mycobiome analysis of multiple cancer types from the TCGA cohort suggested that BRAC may be associated with *M. globosa* (8). Other recent data concluded that immune responses due to the presence of intratumor-growing fungi may influence intratumor inflammation, leading to a reduction in OS of BRAC patients (9). While these studies provide an explicit link between *M. globosa* colonization and BRAC development, more research is needed to understand the underlying mechanisms by *M. globosa* promotes BRAC progression.

DMBA-induced breast tumor in animals is a well-accepted experimental carcinogenesis model similar to human BRAC (31). To better understand the role of the microbiome in tumorigenesis *in vivo*, we have analyzed the effect of *M. globosa* on DMBA-induced tumorigenesis. *M. globosa* was implanted at a mammary fat pad in mice with DMBA-induced BRAC. Our results demonstrated that the abundance of *M. globosa* in DMBA-induced BRAC tissues was higher than that in normal mammary glands (Fig. 1C). Meanwhile, colonization with *M. globosa* reduced the survival of mice (Fig. 1B). Additionally, after 8 and 16 weeks, the incidence of tumors in the DMBA + *M. globosa* group was higher than in the DMBA group, which also means a higher risk of tumorigenesis in mice with *M. globosa* infection (Fig. 1D). Then, AMPB was used to ablate *M. globosa*, and the result showed that the content of *M. globos*a cells and tumor incidence was reduced but had no significant effect on OS (Fig. 1E and F). To determine the effect of fungal dysbiosis on the progression of BRAC, we used oral AMPB to ablate the fungi in orthotopic tumor transplantation in the 4T-1 mice model. *M. furfur*, *C. albicans*, *S. cerevisae*, and VEH were used as control experiments. Intriguingly, we found significant changes in tumor volume and weight after *M. globosa* injection into the breast fat pad, whereas injection with the other fungi had no significant effect on tumor growth (Fig. 2B through E). In addition, the result showed MCF-7 cell proliferation using a transwell assay in the co-culture system, which is possibly caused by metabolites of *M. globosa* (Fig. S1). The metabolites of the potent aryl hydrocarbon receptor (AhR) ligands produced by *Malassezia* isolates have been related to the pathogenic potential of this yeast (32). Our result showed that *M. globosa* infection of MCF-7 cells induces activation of the AhR, but similar results were found in *M. furfur* stimulated MCF-7 cells (Fig. S4). The overexpression of AhR may be one of the factors that promote MCF-7 cells by *M. globosa*, but it is not the only one. Injection of AMPB or FLU in suitable concentrations had an inhibitory effect on tumors in 4T-1 orthotopic transplantation mice, which means the elimination of *M. globosa* can inhibit tumor growth (Fig. S2). Meanwhile, treatment with FLU and DOX showed lower tumor volume compared with the DOX group, which proved fungal ablation enhanced the effect of DOX chemotherapy (Fig. 2G through K).

Epidemiological studies have shown that chronic inflammation predisposes individuals to various cancers (33, 34). In recent years, an increased number of studies have demonstrated that pathogenic microorganisms can promote tumor growth (35). Intertumoral microbiota is involved in tumor-promoting inflammation (36). Notably, mucosal tissue with microbial colonization appears to be the main cause of cancers with IL-17A overactivation (20). The current understanding of IL-17A is that it is critical in inflammation and acting against infectious disease (37). Previous studies showed that IL-17A promotes BRAC invasion, which may indicate a pro-tumor effect of inflammation (38). Therefore, we suspect that the presence of *M. globosa* may lead to the overexpression of IL-17A and chronic inflammation, which ultimately promotes cancer progression. In our study, the levels of IL-17A correlated with BRAC progression in mice with *M. globosa* colonization. The IL-17A levels increased significantly in the *M. globosa* colonized group compared with the VEH group (Fig. 3B and C). In line with the level of the *M. globosa*-induced IL-17A cytokine response upregulation, the expression of the key instructing factors IL-1β, IL-6, IL-23, and TNF-α was upregulated in the *M. globosa* colonized mice compared to the VEH group ( Fig. S3D and E), which may lead to chronic inflammation within the TEM and thus accelerate tumor growth. The results of those inflammatory cytokine levels in the MCF-7 cells (Fig. S3G) and 4T-1 cells (Fig. S3H) were consistent with those done in mice.

TME plays an important role in cancer development and metastasis (39). Among multifarious immune cells, macrophages play an important role in the recognition and elimination of bacteria, fungi, and viruses (40). Since numerous studies demonstrated that the microbiome could promote cancer progression via suppressing anti-tumor immunity, our data unraveled that the abundance of *M. globosa* positively correlated with the number of macrophages *in vivo* (Fig. 3D). Previous studies reported that IL-17A can promote the recruitment of macrophages and induce their cytokine/chemokine production (41, 42). Herein, RNA sequencing unraveled that *M. globosa* could shape the immune responses of the host. We also found that macrophages and IL-17A were highly expressed in *M. globosa* colonization tumor tissues using IF. Co-localization of IL-17A and F4/80 macrophages showed that IL-17A was highly expressed in macrophages, which means the IL-17A can recruit macrophages leading to tumor growth (Fig. 3F).

In TME, chemokines and growth factors secreted by tumors could induce monocytes to recruit and differentiate into macrophages. TAMs undergo polarization into M1 and M2 phenotypes. M1 macrophages release a range of pro-inflammatory cytokines to impede the growth of tumors, while M2 macrophages produce anti-inflammatory cytokines to facilitate tumor development and suppress the immune response (43). To explore the effect of *M. globosa* activation on macrophage phenotypes, we demonstrated that macrophages exhibited the M2 phenotype after being stimulated with *M. globosa in vitro*, suggesting that *M. globosa* promotes the progression of BRAC via promoting M2 polarization of macrophages. The levels of CD206 and CD86 were much higher in macrophages stimulated with *M. globosa* at 12 and 24 h compared with the non-*M*. *globosa*-infected group (Fig. 4A through C). After direct stimulation of THP-1-derived macrophages by *M. globosa*, chemokines of M2 and M1 were both upregulated (Fig. 4F through K). Furthermore, the inflammatory factors produced by *M. globosa-*stimulated MCF-7 cells may have a dominant role in the polarization of macrophages. Therefore, we co-cultured *M. globosa* with MCF-7 cells, and the supernatant medium was used to stimulate THP-1-derived macrophages. In this study, M1- and M2-like markers were upregulated, with M2-like markers being dominant when THP-1 cells were co-cultured with the supernatant medium from *M. globosa* and MCF-7 cells (Fig. 4F through K). TLR4 could sense pathogen-associated molecular patterns derived from microbiota (44). Meanwhile, the TLR4 orchestrates downstream Nf-κB activity and regulates the production of inflammatory cytokines (45). In this section, we demonstrated that *M. globosa* could promote M2 polarization in a TLR4/Nf-κB dependent way (Fig. 4D and E). These results suggested that *M. globosa* can modulate TAM to assume an M2-like phenotype, thereby facilitating immune surveillance evasion and promoting angiogenesis.

Although the link between fungi and IL-17A secretion remains to be determined, this study reveals an association between the intertumoral presence of *M. globosa* and the IL-17A/macrophages axis in BRAC TME. A transwell assay verified that rhIL-17A can promote the proliferation of THP-1, which means that IL-17A may promote the development of breast tumor cells through macrophage attraction (Fig. 3G). Thus, the depletion of IL-17A was conducted in mice, and the results demonstrated that the tumor volume was reduced in the anti-17A + *M. globosa* group compared with *M. globosa* colonization (Fig. 5A through C). Previous reports demonstrated that tumor cells treated with IL-17A induce M2 polarization in RAW264.7 and THP-1 cells, possibly as a result of the COX-2/prostaglandin E2 (PGE2) pathway (46). In our study, we found that the expression of CD86 and CD206 at the mRNA level was greatly enhanced after stimulation with *M. globosa*, while it was decreased after IL-17A blockade (Fig. 5D through E). Meanwhile, the results showed a reduction of M2 after local IL-17A neutralization (Fig. 5F). We infer that M2 polarization could be driven by *M. globosa* promoting the activation of IL-17A leading to attracted macrophages and modification of macrophages into an immunosuppressive phenotype, which may contain a positive feedback loop.

It has been reported that the lipid dependence of *M. globosa* is due to the lack of the gene coding for fatty acid synthase (47, 48). Therefore, *M. globosa* requires an external source of lipids for its growth. We hypothesized that *M. globosa* utilizes these lipid sources for its development since the breast is mainly composed of adipose tissue and fibro-glandular tissue. This may result in a large set of metabolites and enzymes at the site of fat pads in the mammary glands of mice. Lipids are circulating as lipoproteins, consisting of unesterified cholesterol, triglycerides, and phospholipids (49). The evidence of *M. globosa* causing lipid disturbance in tumor tissue is consistent with the results of the oil red O stain and the levels of TG, LDLC, and HDLC (Fig. S5G). The oil red O staining showed that the number of lipid drops increased in the *M. globosa*-induced group, but there was no significant change after being stimulated with *S. cerevisiae* (Fig. S5A). However, the content of non-esterified fatty acid (NEFA) was significantly upregulated following infection with *M. globosa* (Fig. S5B). With the help of lipases, *M. globosa* releases metabolites, such as AA and other triglycerides, which act as immune stimulatory molecules (28). COX-2 and 5-LOX are released from phospholipids primarily by fatty acid amide hydrolase as substrates of AA (50). The levels of COX-2 and 5-LOX increased after being infected with *M. globosa* (Fig. S5C through E). Furthermore, RNA sequencing analysis showed the overexpression of Sphk1 during *M. globosa*-induced BRAC (51). Sphk1 are lipid kinases and play a role outside and inside the cell to regulate biological processes (52). To further validate the relationship between *M. globosa* and BRAC, we established a co-culture model of *M. globosa* and MCF-7 transfected with Sphk1-siRNA. Reduced expression of Sphk1 leads to the reduction of intracellular lipids and the levels of TG, HDLC, and LDLC, further alleviating the stimulatory effect of *M. globosa* on MCF-7 (Fig. 6H and I). Previous research demonstrated that Sphk1 promoted the secretion of inflammatory cytokines in lipopolysaccharide-stimulated RAW264.7 cells by activating the Nf-κB and STAT3 signaling pathways (53). In addition, Qu et al. showed that the content of IL-6, IL-1β, and TNF-α significantly increased after Sphk1 overexpression (54). In this study, we found that knockdown of Sphk1 in MCF-7 cells after stimulation with *M. globosa* decreased the expression of the STAT3/Nf-κB signaling pathway and reduced the content of IL-6, IL-1β, and TNF-α (Fig. 6J through L), which suggested that downregulation of Sphk1 also alleviate chronic inflammation and cell migration in TME.

Although there are some valuable findings in this work, its limitations should not be ignored. It is noteworthy that *M. furfur* colonization does not significantly affect tumor proliferation. Ramírez et al. showed that different *Malassezia* species have a preference for accumulating certain lipid sources. For example, undetectable traces of fatty acids and hydroxyl fatty acids occurred in *M. globosa*, but these can be found in *M. furfur* (55). We observed the contents of lipase (LPS), NEFA, and TG in tumor tissue and found that the content of TG in the *M. globosa* group was higher than that of the *M. furfur* group, but NEFA and LPS amounts did not differ between the *M. globosa* and *M. furfur*-treated groups (Fig. S4). We suspect that this relates to genetic and phenotypic variation and biological differences between the various *Malassezia* species. This work mainly focused on the relationship between IL-17A and macrophages but ignored other immune cell subtypes. Thus, our further work will explore the role of Th17 cells in *M. globosa*-promoting BRAC.

In conclusion, our work demonstrated that *M. globosa* colonization promoted BRAC progression in mice models. Those data suggest that IL-17A-activated BRAC attracts macrophages to TME during *M. globosa* colonization. Meanwhile, the attracted macrophages polarized into M2-like TAMs, which finally induced an immunosuppressive microenvironment to promote tumor development. Furthermore, *M. globosa*-colonized BRAC leads to overexpression of Sphk1. Knockdown of Sphk1 can inhibit cellular lipid content and cell proliferation of MCF-7 cells.

## MATERIALS AND METHODS

### Mice

Female BALB/c mice were obtained from Charles River (Beijing, China). The mice were housed in a specific pathogen-free environment on a 12 h light/dark cycle with food and water *ad libitum*.

### Fungi and cell lines

*M. globosa* (HX19), *M. furfur* (CBS 1817), *C. albicans* (SC5314), and *S. cerevisiae* (BY4741) were used in this experiment. In growth assays, strains were streaked from frozen glycerol stocks onto modified Dixon media agar (mDixon) and Sabouraud dextran broth after 3 days of incubation at 37°C. A single colony was suspended in sterile distilled water to an optical density at 600 nm (OD600) of 0.7 and diluted as appropriate for the assay.

MCF-7 (catalog: FH0215), 4T-1 (catalog: FH0366), and THP-1 (catalog number: FH0112) were purchased from Shanghai Fuheng Biotechnology Co., Ltd. (Shanghai, China). 4T-1 and THP-1 cell lines were maintained in Roswell Park Memorial Institute (RPMI) 1640 medium supplemented with 10% fetal bovine serum (FBS) and penicillin at 100 U/mL. MCF-7 cell line was maintained in Dulbecco’s modified eagle medium (DMEM) supplemented with 10% FBS and penicillin at 100 U/mL. Cells were incubated at 37°C in 5% CO_2_ and 95% air atmosphere. Cells were grown to passage 30 before discarding. BRAC cell lines (MCF-7 and 4T-1) and THP-1-derived macrophages were stimulated with *M. globosa* (MOI = 10).

### CCK-8 and XTT assay

MCF-7 cells and 4T-1 cells (1–5 × 10^3^ cells/mL) were placed on 96 well plates. The cells were treated with Tween 80 (0.01%, 0.05%, 0.1%, 0.5%, and 1%) or glucose (0.25%, 0.5%, 1%, 2%, and 4%) for 24 h, and cell viability was determined with the CCK-8 test (Beyotime, Shanghai, China). In addition, an *M. globosa* and *S. cerevisiae* cell suspension was incubated for 24 and 48 h at 37°C in a 96-well plate. The proliferation of *M. globosa* and *S. cerevisiae* was analyzed using the 2, 3-bis (2-methoxy-4-nitro-5-sulfophenyl)-5-[(phenylamino) carbonyl]-2H-tetrazolium hydroxide (XTT)-reduction assay (Beyotime, Shanghai, China).

### Migration assay

Costar 24-transwell plates with 0.22 µm polycarbonate membranes were used to measure cell migration and invasion ability of MCF-7 and 4T-1 cells. Cells were harvested and re-suspended in an FBS-free RPMI 1640 medium, and 1 × 10^3^ cells were added to the upper chamber. *M. globosa* cells were re-suspended in RPMI 1640 medium with 0.05% Tween 80, and then the *M. globosa* suspension was added to the lower chamber. After 24 h, the cells that had traversed the polycarbonate membrane were fixed with 4% paraformaldehyde for 15 min, stained with 1% crystal violet solution for 20 min, and then washed with PBS. The non-traversed cells were gently wiped off with a cotton swab, and the number of traversed cells was counted under the light microscope.

### The DMBA-induced mouse breast carcinogenesis model

Female BALB/c mice were randomly divided into four groups (*n* = 10): control group (CON), DMBA-induced group (DMBA), *M. globosa* colonized group (DMBA + *M. globosa*), and antifungal group (DMBA + *M. globosa* +AMPB). After adaptive feeding, all groups (except CON) were gavaged with DMBA (30 mg/kg) once a week lasting for 16 weeks. The mice in the DMBA + *M. globosa* group and DMBA + *M. globosa* + AMPB group were injected with *M. globosa* suspension (1 × 10^8^ CFU/mL) in the mammary fat pad three times a week, while the mice in the DMBA + *M. globosa* + AMPB group were applied with AMPB (1 mg/mL) by oral gavage three or four times a week.

### Tumor model and treatment protocol

To generate tumors, 4T-1 cells, which are syngeneic to BALB/c, were injected into the mammary gland. The growth of tumors was monitored from the third day after 4T-1 cells inoculation, and the size of palpated tumors for each mouse individually was recorded. 4T-1 cells were harvested and washed in a serum-free medium and resuspended in PBS. Cells in 0.1 mL PBS were implanted into the female BALB/c mice in the abdominal mammary fat pad.

To ablate the mycobiome, animals were administered AMPB (1 mg/mL) by subcutaneous injection for 5 consecutive days (4). Female BALB/c mice were orthotopically (mammary fat pad) injected with 4T-1 (1 × 10^6^ cells/mL) tumor cells after commencing treatment with APMB. When the tumor reached approximately 500 mm^3^ in size, mice were injected with *M. globosa* cells in a mammary fat pad. For species-specific repopulation experiments, *M. globosa* (1 × 10^8^ CFU/mL), *M. furfur* (1 × 10^8^ CFU/mL), *S. cerevisiae* (1 × 10^8^ CFU/mL), *C. albicans* (1 × 10^8^ CFU/mL), and VEH) were used to gavage mice following fungal ablation with APMB orally. The mice were immediately euthanized when the maximal tumor limit has been exceeded 1,200 mm^3^. During autopsy mice, each primary tumor was isolated, measured, and weighed on digital scales. The volume of tumors was calculated as follows:


$$v=\frac{length\times width^{2}}{2}$$

### AMPB or FLU treatment protocol

BALB/c mice were divided into three groups (*n* = 5): *M. globosa* colonized group (*M. globosa*), FLU treatment group (*M. globosa* + FLU), and AMPB treatment group (*M. globosa* + AMPB). To generate tumors, 4T-1 cells, which are syngeneic to BALB/c, were injected into the mammary gland. To ablate the *M. globosa* in mice, AMPB (1 mg/mL) was administered to mice by oral gavage twice a week, and then the mice were fed with 0.5 µg/mL AMPB in drinking water for the duration of the experiment. Meanwhile, the mice in the *M. globosa* + FLU group were fed with 0.5 mg/mL FLU in drinking water for the duration of the experiment. The tumor tissue was harvested at the end of the experiment.

### Quantitative PCR

Trizol was used to extract RNA from tumor tissue. Reverse transcription was performed in a volume of 20 µL containing 1 µg of total RNA. cDNA synthesis was performed using the ReverAid First Strand cDNA Synthesis Kit (A5006, Promega, USA). The expression of CXCL13, CCL1, CCL2, TGF-β, VEGF, iNOS, and Gapdh in *M. globosa*-stimulated THP-1-derived macrophages and the expression of CD86 and CD206 in *M. globosa* colonization tumor tissue were quantitatively detected by qPCR, and the relative quantitative analysis of the data was carried out by the 2^-∆∆CT^ method. The primer sequences used are shown in Table 1, and the qPCR conditions were as follows: 40 cycles at 95°C for 1 min, 95°C for 15 s, and 55°C for 1 min.

**TABLE 1:** Primers for quantitative qPCR analysis

Gene	Species	Forward primer (5′−3′)	Reverse primer (5′−3′)
*Sphk-1*	Human	ACCACCATCATCGACACCTTC	AAAGGTTGCCAACTGTGCTTC
*CXCL13*	Human	TTGTGATCTGGACCAAGATGAA	GACTTTTGCTTTGGACATGTCT
*VEGF*	Human	ATCGAGTACATCTTCAAGCCAT	GTGAGGTTTGATCCGCATAATC
*TGF-*β	Human	CCAAGTGCTGCCGTCATTTTC	GGCTCGCAGGGATGATTTCAA
*CCL1*	Human	TTGCTGCTAGCTGGGATGT	CTGGAGAAGGGTACCTGCAT
*CCL2*	Human	TTCACTGGCAAGATGATCCC	TGCTTGAGGTGGTTGTGGAA
*iNOS*	Human	TTCAAGACCAAATTCCACCAC	ATTCTGCTGCTTGCTGAGGT
*Gapdh*	Human	GGAGCGAGATCCCTCCAAAAT	GGCTGTTGTCATACTTCTCATGG
*CD206*	Mouse	ACACAAACTGGGGGAAAGGTT	TCAAGGAAGGGTCGGATCG
*CD86*	Mouse	TGTTTCCGTGGAGACGCAAG	TTGAGCCTTTGTAAATGGGCA
*5-LOX*	Mouse	CCATCACCCACCTTCTGC	CACCTGGTCGCCCTCGTA
*COX-2*	Mouse	TTGCTGGCAGGGTTGCTGGTGGTA	CATCTGCCTGCTCTGGTCAATGGAA
*Gapdh*	Mouse	AGGTCGGTGTGAACGGATTTG	GGGGTCGTTGATGGCAACA
*M.gl*	Fungus	CAATAAGTGTGTCTCTGCGG	TTCGCTGCGTTCTTCATCGA
*ITS*	Fungus	GGATCATTAGTGATTGCCTTTATA	TCCTCCGCTTATTGATATG

### Histology

Mouse tumor tissues were fixed in 10% formalin for 24 h and embedded in paraffin. Sections (4 µm) were stained with hematoxylin and eosin and observed using an optical microscope.

### Enzyme-linked immunosorbent assay

Tumor tissue was collected from the animals at the end of the study, stored at –80°C, and used for cytokine measurements by ELISA. IL-1β, IL-6, IL-17, and TNF-α were measured using an ELISA kit following the manufacturer’s protocol (MSK, Wuhan, China), and the plate was read immediately using a microplate spectrophotometer at 450 nm.

### Immunohistochemistry and immunofluorescence

Specimens from mice were formalin-fixed, sectioned, and embedded into paraffin for immunohistochemistry. After blocking unspecific sites with 5% bovine serum albumin, tissues were stained with primary anti-IL-17A (diluted at 1:800), anti-COX2 (diluted at 1:500), or anti-5-LOX (diluted at 1:2,000). A secondary antibody was used and developed with 3,3ʹdiamino benzidine and counterstained with hematoxylin.

IF staining was performed as described previously. For THP-1-derived macrophages, cells were seeded on 14 × 14 mm coverslips, incubated for 24 h, and stimulated with or without *M. globosa* for 24 h. Next, the cells were washed with PBS, fixed with cold 4% formaldehyde for 20 min, and permeabilized in chilled 0.1% Triton X-100 for 2 min. For BRAC tissue, the antigen was extracted by citric acid buffer (pH = 6.0). Primary antibodies used were as follows (Table 2): anti-CD86 (diluted at 1:500), anti-CD206 (diluted at 1:500), anti-F4/80 (diluted at 1:500), and anti-IL-17A (diluted at 1:800). The primary antibodies were detected with fluorescein isothiocyanate isomer I (FITC; diluted at 1:200) or cyanine 5 (Cy5, diluted at 1:200) conjugated goat anti-rabbit IgG, respectively. Nuclei were stained with 4,6-diamino-2-phenyl indole, and IF images were obtained using a super-resolution confocal laser scanning microscope.

**TABLE 2:** Antibodies used in this study

Antibody	Cat#	Manufacturer
Anti-CD86	GB115630	Servicebio
Anti-CD206	GB113497	Servicebio
Anti-F4/80	GB113373	Servicebio
Anti-TLR4	A5258	ABclonal
Anti-AhR	A4000	ABclonal
Anti-Nf-κB	GB11997	Servicebio
Anti-STAT3	A16975	ABclonal
Anti-COX-2	GB11077	Servicebio
Anti-5-LOX	GB111330	Servicebio
Anti-IL17A	GB11110	Servicebio
Anti-Sphk1	A22093	ABclonal
HRP-labeled goat anti-rabbit IgG	GB23303	Servicebio
Anti-GAPDH	GB11002	Servicebio
Cy3 conjugated goat anti-rabbit IgG	GB21303	Servicebio
FITC conjugated donkey anti-rabbit IgG	GB22403	Servicebio
Recombinant human IL-17A	RP00212	ABclonal
Recombinant mouse IL-17A	RP02520	ABclonal
InVivoMAb anti-mouse IL-17A	BE0173	Bio Xcell

### RNA sequencing

RNA-seq assay was performed by BGI Genomics Co., Ltd. The RNA was extracted from the BRAC tumor in the VEH and *M. globosa* group. The RNA extraction method used here was as described above. The edgeR (https://bioconductor.org/packages/release/bioc/html/edgeR.html) was used to analyze the differentially expressed genes with the following filter criteria: *q* value of ≤0.05 and fold change of ≥2.

### Western blot

BRAC tissues and cells were ground, while frozen and radio immunoprecipitation assay were lysed in phenyl methane sulfonyl fluoride containing protease and phosphatase inhibitors and then centrifuged at 12,000 × *g* for 10 min at 4°C. The supernatant was taken, and protein samples were detected by the BCA Protein Assay Kit (Beijing Solarbio Science and Technology Co., Ltd., China, Catalog: PC0020). The separated protein bands were fixed on the polyvinylidene fluoride (PVDF) membrane (Millipore Sigma, Inc., Catalog: IPVH00010). The membrane was then sealed with skimmed milk and then reacted overnight at 4°C with rabbit anti-Nf-κB (diluted at 1:1,000), rabbit anti-TLR4 (diluted at 1:1,000), and rabbit anti-Gapdh (diluted at 1:3,000), respectively. After washing three times with tris-buffered saline and Tween 20 (TBST) for 5 min, the HRP-labeled goat anti-rabbit IgG (diluted at 1:1,000) was incubated at room temperature for 60 min and then washed with TBST for 5 min and detected with the chemiluminescence system Quantum HRP substrate.

### Oil red O staining

The oil red O staining assay was used to assess the presence/abundance of intracellular lipid droplets. MCF-7 cells and 4T-1 cells were cultured in 24-well plates with and without *M. globosa* at an MOI = 10. After 24 h of incubation, cells were fixed with 4% paraformaldehyde for 15 min. MCF-7 cells and 4T-1 cells were incubated with oil red O stain for 20 min and rinsed with PBS.

### Sphk1-siRNA transfection

The MCF-7 cells were placed into 12-well plates with 1 mL of DMEM. Sphk1-siRNA and negative controls (20 µM) were mixed with 200 µL Opti-MEM (Thermo Fisher Scientific, USA, catalog: 31985070) to reach a final concentration of 50 nM. We optimized the Sphk1-siRNA: transfection reagent ratio between 20:1 and 5:1 (nM:μL) to enhance transfection efficiency. siRNA-Mate Transfection Reagent (Lablead, China, catalog: TR002) was used to treat MCF-7 cells following the instructions provided by the manufacturer. After 48 h, the most effective Sphk1-siRNA interference fragment was chosen based on protein assessment. The transfected MCF-7 cells were then cultured for subsequent experiments.

### Measurement of lipid content

For BRAC tissue, 0.1 g samples of tissues were prepared into a 10% (wt/vol) buffered homogenate after rinsing in ice-cold isotonic saline. For the MCF-7 cell, cells were cultured at a density of 1 × 10^6^ cells/well in six-well plates. Then, MCF-7 cells and tissue were washed with cold PBS and centrifuged at 12,000 g for 10 min. The supernatant was removed, and the precipitate was sonicated. According to the introductions of these kits purchased from Thermo Fisher Scientific Inc., these kits were used to measure the levels of contents of NEFA (catalog: EEA017), LDLC (catalog: EEA014), HDLC (catalog: EEA012), and TG (catalog: EEA028) in BRAC tissue and cells.

### Data analysis

Statistical analysis was performed using GraphPad Prism 8 (GraphPad Software, Inc.). Data were expressed as mean ± SD. Student’s *t* test (two-tailed) was utilized to compare two groups. One-way analysis of variance (ANOVA) using the Tukey post-test was utilized when more than two groups were compared. The results were significant when ***P < 0.005; **P < 0.01; *P < 0.05.

## Data Availability

RNAseq data that support the findings of this study have been deposited in NCBI BioProject with the primary accession code PRJNA1138454 and in NCBI Sequence Read Archive (SRA) with the primary accession code SRP521520.

## SUPPLEMENTAL MATERIAL

Figure S1 mbio.01993-24-s0001.tif *M. globosa* promotes the proliferation of BRAC cells.

Figure S2 mbio.01993-24-s0002.tif Efficacy of anti-fungal treatments in BRAC.

Figure S3 mbio.01993-24-s0003.tif *M. globosa* elicits an inflammatory response from TEM.

Figure S4 mbio.01993-24-s0004.tif AhR translocates to the nucleus of MCF-7 upon stimulation with *M. globosa* and *M. furfur*.

Figure S5 mbio.01993-24-s0005.tif Lipid accumulation occurs in colonization with *M. globosa*.

Legends mbio.01993-24-s0006.docx Supplemental figure legends.

Table S1 mbio.01993-24-s0007.xlsx Results of the differential gene expression analysis performed.

Table S2 mbio.01993-24-s0008.xlsx KEGG analysis of breast tumor incubation with *M. globosa*.

Table S3 mbio.01993-24-s0009.xlsx GO analysis of breast tumor incubation with *M. globosa*.

Table S4 mbio.01993-24-s0010.xlsx Estimation matrix of immune infiltrates.

ASM does not own the copyrights to Supplemental Material that may be linked to, or accessed through, an article. The authors have granted ASM a non-exclusive, world-wide license to publish the Supplemental Material files. Please contact the corresponding author directly for reuse.
