# Supplementary figures and images for: Breast cancer colonization by Malassezia globosa accelerates tumor growth
Source: mBio. 2024 Sep 5;15(10):e01993-24. doi: 10.1128/mbio.01993-24 (PMC11481877; doi:10.1128/mbio.01993-24)

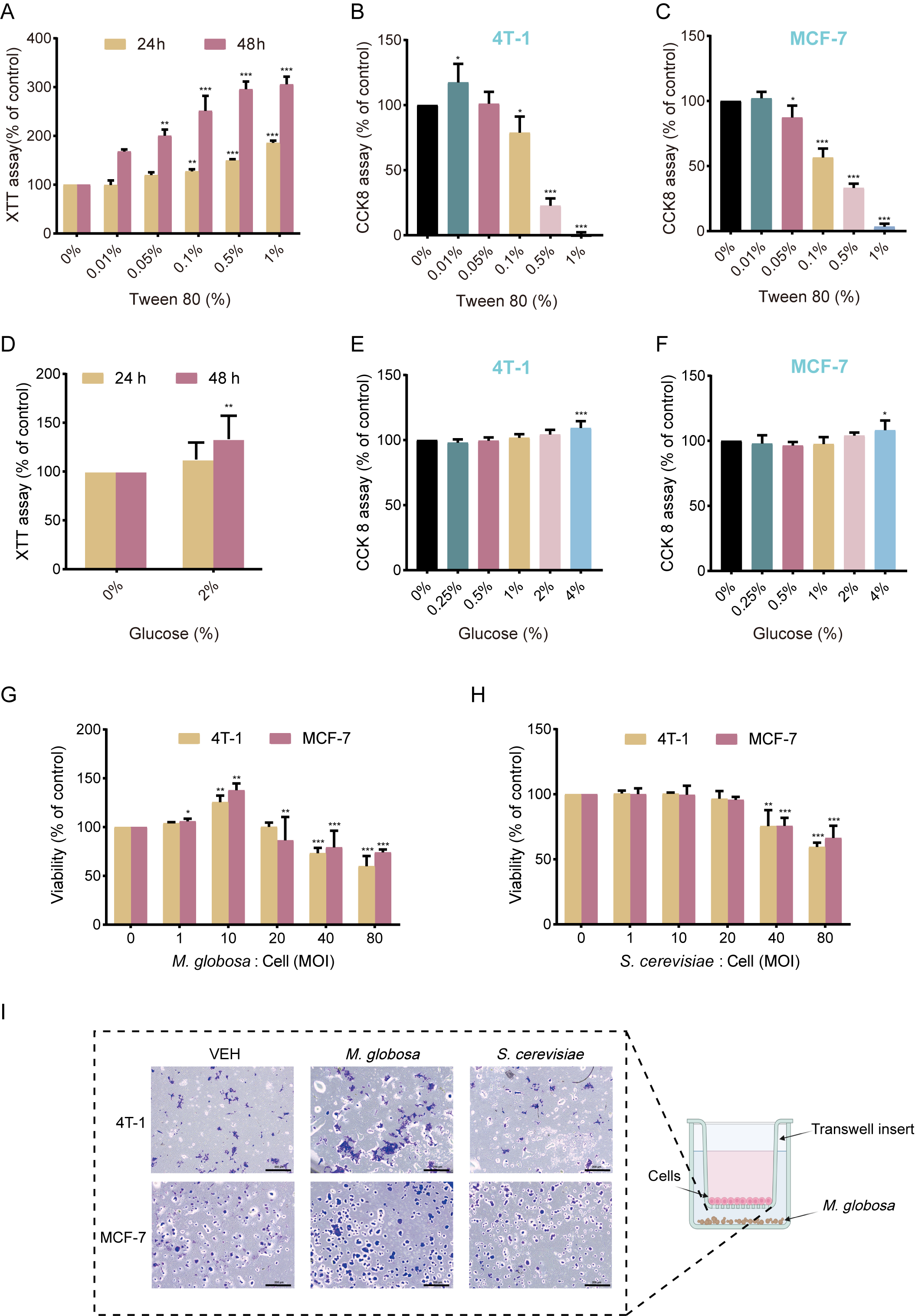

Supplement: Figure S1 — M. globosa promotes the proliferation of BRAC cells. [file mbio.01993-24-s0001.tif]

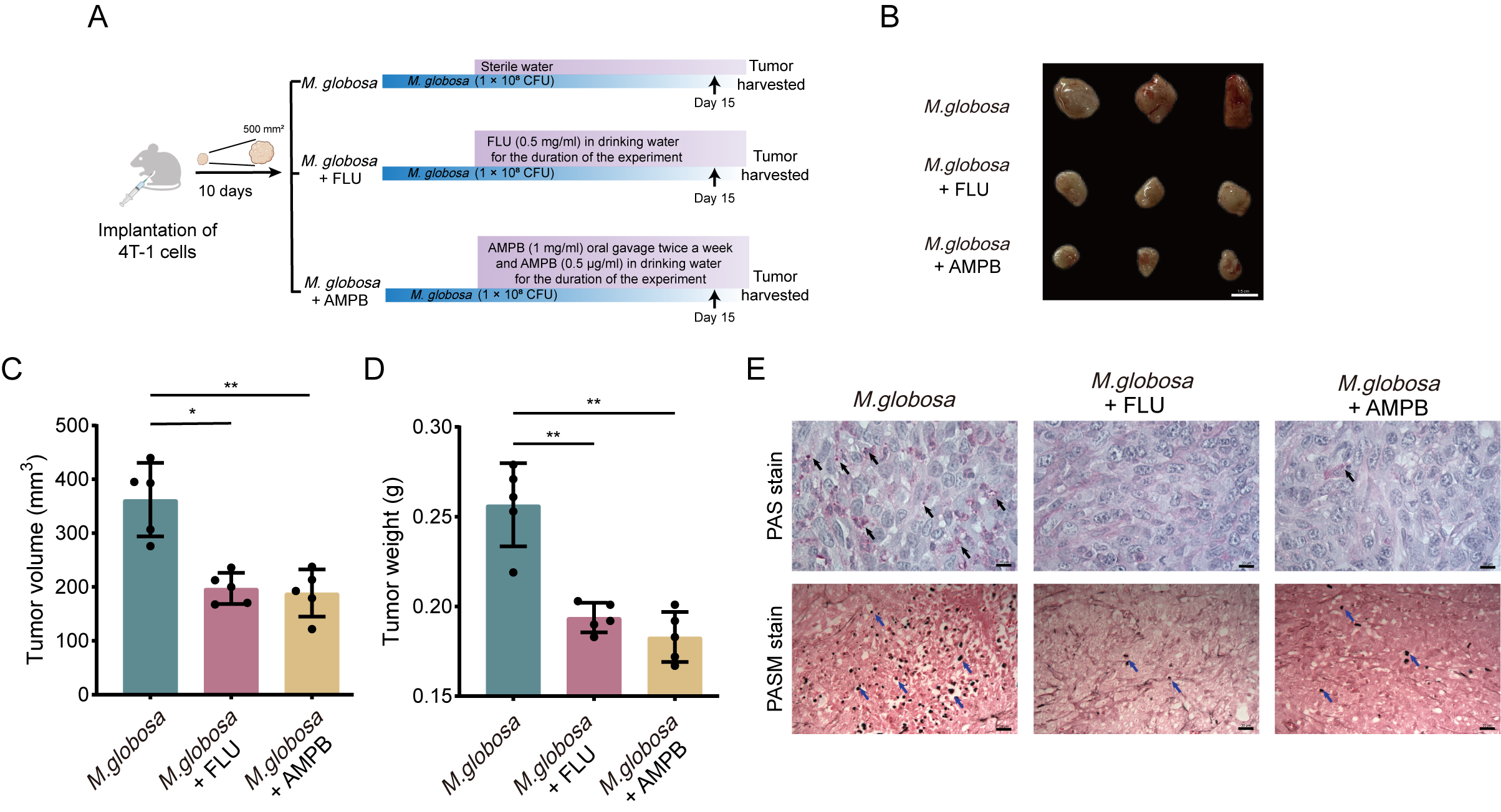

Supplement: Figure S2 — Efficacy of anti-fungal treatments in BRAC. [file mbio.01993-24-s0002.tif]

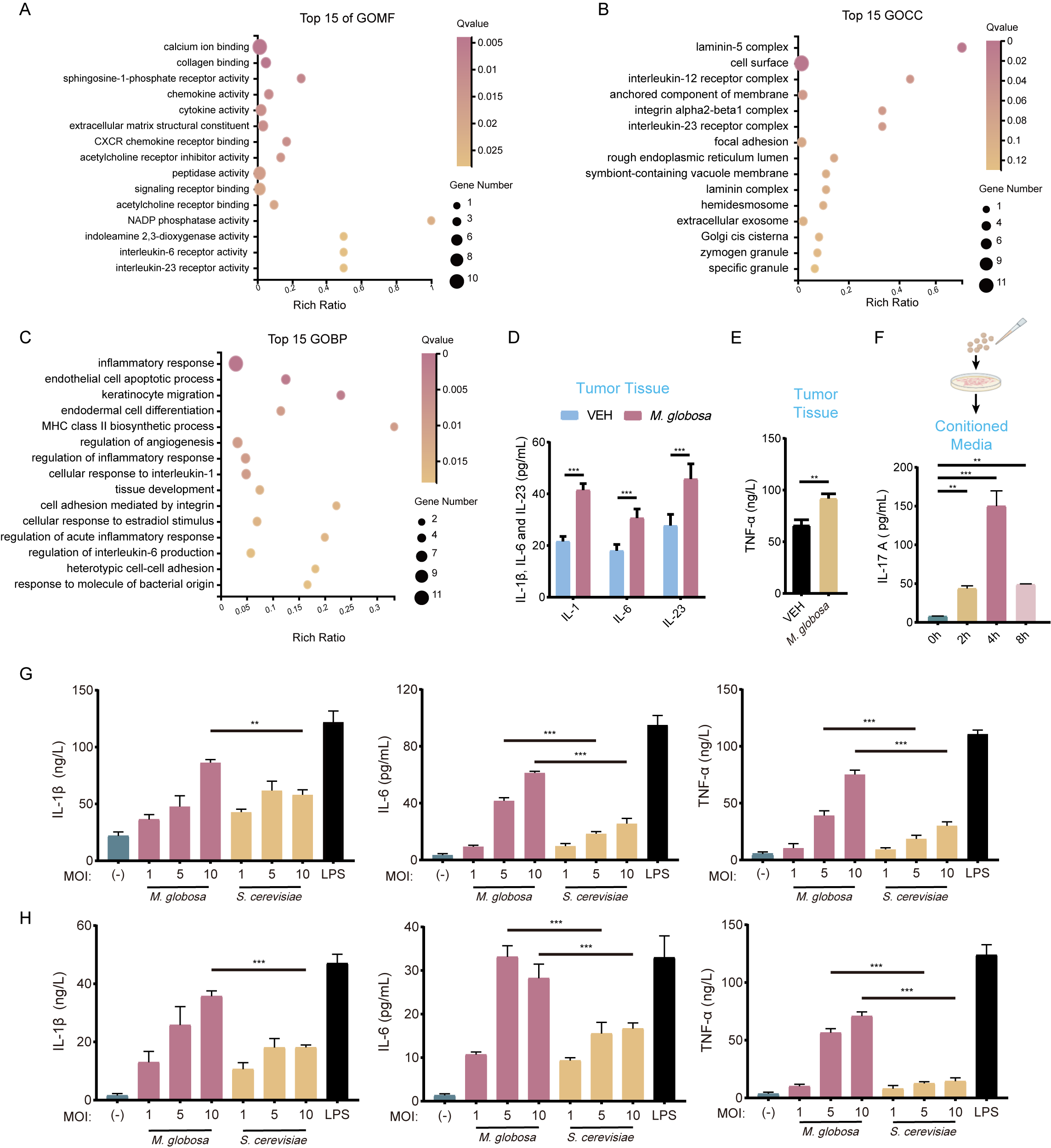

Supplement: Figure S3 — M. globosa elicits an inflammatory response from TEM. [file mbio.01993-24-s0003.tif]

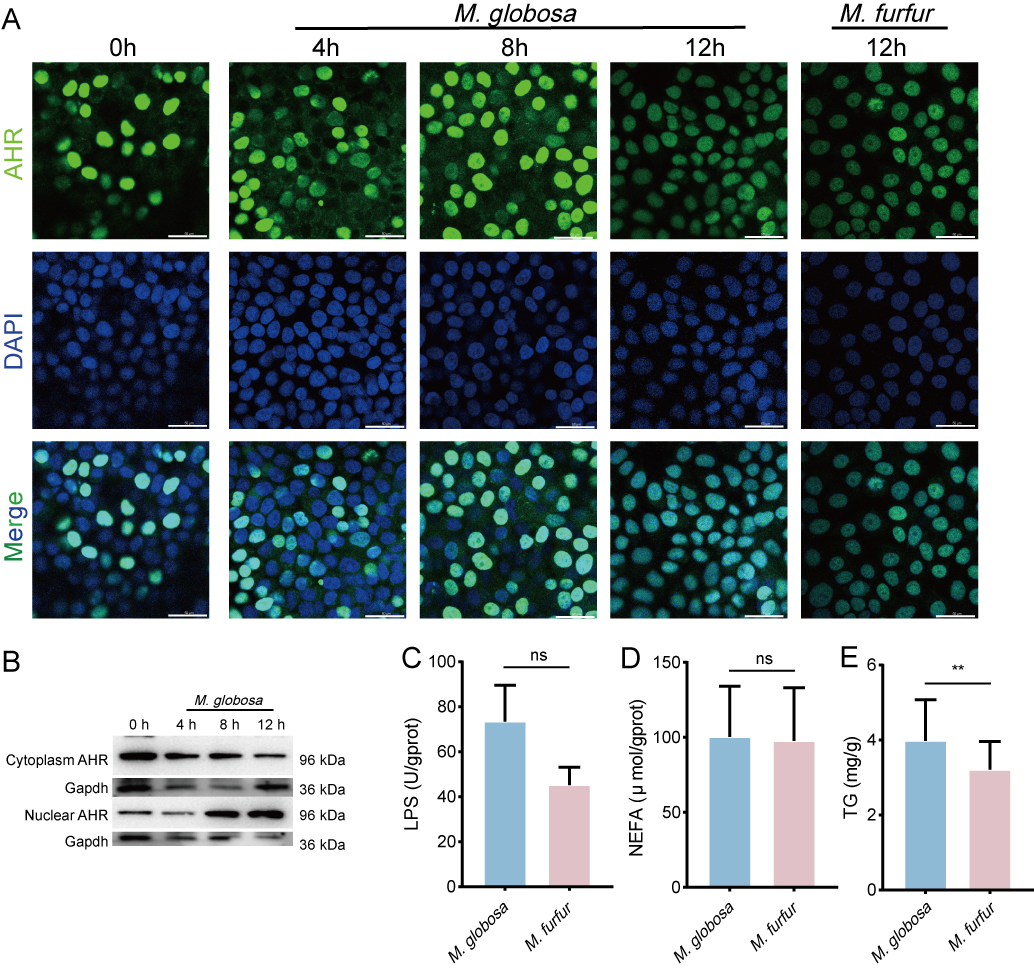

Supplement: Figure S4 — AhR translocates to the nucleus of MCF-7 upon stimulation with M. globosa and M. furfur. [file mbio.01993-24-s0004.tif]

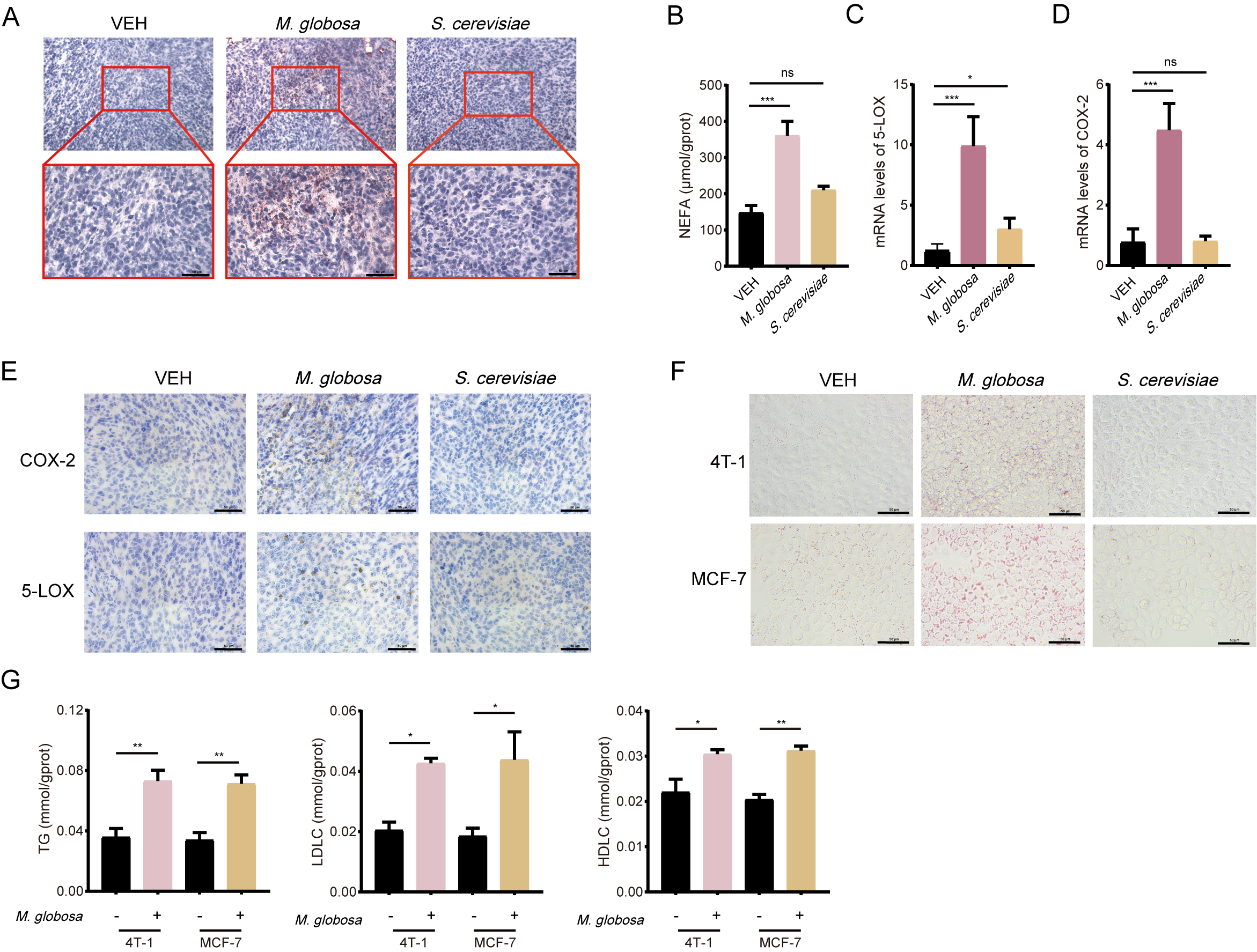

Supplement: Figure S5 — Lipid accumulation occurs in colonization with M. globosa. [file mbio.01993-24-s0005.tif]
