# Supplementary material for: Breast cancer colonization by Malassezia globosa accelerates tumor growth
Source: mBio. 2024 Sep 5;15(10):e01993-24. doi: 10.1128/mbio.01993-24 (PMC11481877; doi:10.1128/mbio.01993-24)
Supplement: Legends — Supplemental figure legends. [file mbio.01993-24-s0006.docx]

**Supplementary Figure Legends**

Supplementary Figure 1. *M. globosa* promotes the proliferation of BRAC cells. (A) Metabolic activity (XTT reduction assay) of *M. globosa* in RPMI 1640 broths with Tween 80. (B-C) The cell viability of 4T-1 and MCF-7 was measured following treatment Tween 80. (D) Metabolic activity (XTT reduction assay) of *S. cerevisiae* in RPMI 1640 broths with glucose. (E-F) The cell viability of 4T-1 and MCF-7 was measured following treatment glucose. (G) Cell viability of 4T-1 and MCF-7 cells was measured following infection with *M. globosa*. (H) Cell viability of 4T-1 and MCF-7 cells was measured following infection with *S. cerevisiae*. (I) Migration of 4T-1 and MCF-7 cells infection with *M. globosa* or *S. cerevisiae* was determined by transwell assay. Scale bars represent 200 μm. Data are mean values ± SD. ^*^*p* < 0.05, ^**^*p* < 0.01, and ^***^*p* < 0.001.

Supplementary Figure 2. Efficacy of anti-fungal treatments in BRAC. (A) Schematic diagram of FLU or AMPB treatment in drinking water for the duration of the experiment. (B) Representative photographs of breast tumor. Scale bars represent 1 cm. (C-D) Weight and volume of tumors after treatment. (E) Representative sections of periodic acid-schiff (PAS) stain and periodic acid-silver methenamine (PASM) stain. Scale bars represent 20 μm. Data are mean values ± SD.  ^*^*p* < 0.05, ^**^*p* < 0.01, and ^***^*p* < 0.001.

Supplementary Figure 3. *M. globosa* elicits an inflammatory response from TEM. (A-C) GOMF, GOCC, and GOBP enrichment analysis showed the top 15 enrichment pathways. (D) The levels of IL-1β, IL-6, and IL-23 in breast tumors. (E) The level of TNF-α in breast tumors. (F) MCF-7 cells were stimulated by *M. globosa* and IL-17A levels were measured in culture supernatants. (G) The levels of IL-1β, IL-6, and TNF-α after stimulation with *M. globosa* in MCF-7 cells. (H) The levels of IL-1β, IL-6, and TNF-α after stimulation with *M. globosa* in 4T-1 cells. Data are mean values ± SD. ^*^*p* < 0.05, ^**^*p* < 0.01, and ^***^*p* < 0.001.

Supplementary Figure 4. Aryl hydrocarbon receptor (AhR) translocates to the nucleus of MCF-7 upon stimulation with *M. globosa* and *M. furfur*. (A) AhR is shown in green, the nuclei are shown in blue. AhR translocation to the nucleus was evident after 4 h and highest at 12 h after stimulation. Scale bars represent 20 μm. (B) Western blotting assays analyzed the expression of AhR after stimulation with *M. globosa*. (C-E) The levels of LPS, NEFA, and TG in MCF-7 cells. Data are mean values ± SD. ^*^*p* < 0.05, ^**^*p* < 0.01, and ^***^*p* < 0.001.

Supplementary Figure 5. Lipid accumulation occurs in colonization with *M. globosa*. (A) Oil red O staining of breast tumor colonization with VEH, *M. globosa*, and *S. cerevisiae*. Scale bars represent 50 μm. (B) Activity of NEFA. (C-D) The mRNA levels of COX-2 and 5-LOX. (E) Representative IHC stain of COX-2 and 5-LOX. Scale bars represent 50 μm. (F) Oil red O staining in 4T-1 and MCF-7 cells. Scale bars represent 50 μm. (G) Activities of TG, LDLC, and HDLC. Data are mean values ± SD. ^*^*p* < 0.05, ^**^*p* < 0.01, and ^***^*p* < 0.001.
